# Osteoblastic Lesions as the First Presentation of a Gastric Mixed-Type Adenocarcinoma: A Case Report

**DOI:** 10.7759/cureus.51121

**Published:** 2023-12-26

**Authors:** Patrícia Amaral de Almeida, André Carvalho, Fátima Guedes, Isabel Bessa, Abílio Gonçalves

**Affiliations:** 1 Internal Medicine, Hospital Distrital da Figueira da Foz, Figueira da Foz, PRT

**Keywords:** gastric tumor, metastatic gastric adenocarcinoma, osteoblastic metastasis, mixed-type gastric adenocarcinoma, osteoblastic lesions

## Abstract

Bone metastasis might be associated with several tumors; however, the association between gastric malignant neoplasms and bone secondary lesions is very rare, with the osteoblastic form having the rarest presentation. In fact, osteoblastic lesions, as the first presentation of gastric adenocarcinomas, are even rarer and known to have a very poor prognosis associated with them.

Therefore, we present a clinical case of a patient with lower back pain as the first symptom, which led to the diagnosis of osteoblastic lesions of the spine and iliac bones, suggested as secondary lesions. Later, the investigation of the primary tumor led to the diagnosis of a gastric adenocarcinoma (stage IV disease).

In this report, we highlight the steps taken for the etiological study course and the challenges associated with them from the beginning. We also emphasize the very unfavorable evolution of our patient, with the inability to carry out targeted treatment, neither curative nor palliative, due to the advanced stage of the disease and the very poor survival time associated with it.

## Introduction

Bone metastasis can be associated with several malignant neoplasms, with osteolytic lesions being more frequent and osteoblastic lesions less common. The neoplasms most frequently associated with osteoblastic metastases are prostate malignant tumors, followed by breast and, although much less frequently, thyroid, colon, pancreas, and bladder neoplasms [1].

Gastric neoplasms very infrequently metastasize to the bone and even more rarely in the form of osteoblastic lesions, with rare reports in the literature. Furthermore, bone metastases, as the first presentation of gastric cancer without previous gastrointestinal symptoms, are very rare and known to have a very poor prognosis associated [1-4].

Thus, the authors present a clinical case in which bone osteoblastic lesions were the first presentation and the leading point to the investigation and diagnosis of a gastric adenocarcinoma. 

This article was previously presented as an oral presentation/communication at the Portuguese Internal Medicine Society’s 28th Internal Medicine National Congress on October 2, 2022.

## Case presentation

A 61-year-old male presented to the emergency department with a history of severe lower back pain that had started one month before and had been progressively worsening since then. The pain had mechanical characteristics, worsening with standing and flexion of the torso, and was partially relieved with opioid treatment. The patient also affirmed that in that period, he had lost 11% of his usual body weight and had had several episodes of nocturnal sweats.

The patient had a medical history of type 2 diabetes mellitus, was controlled with metformin 1 gram (g) daily and had benign prostatic hyperplasia.

**Figure 1:**
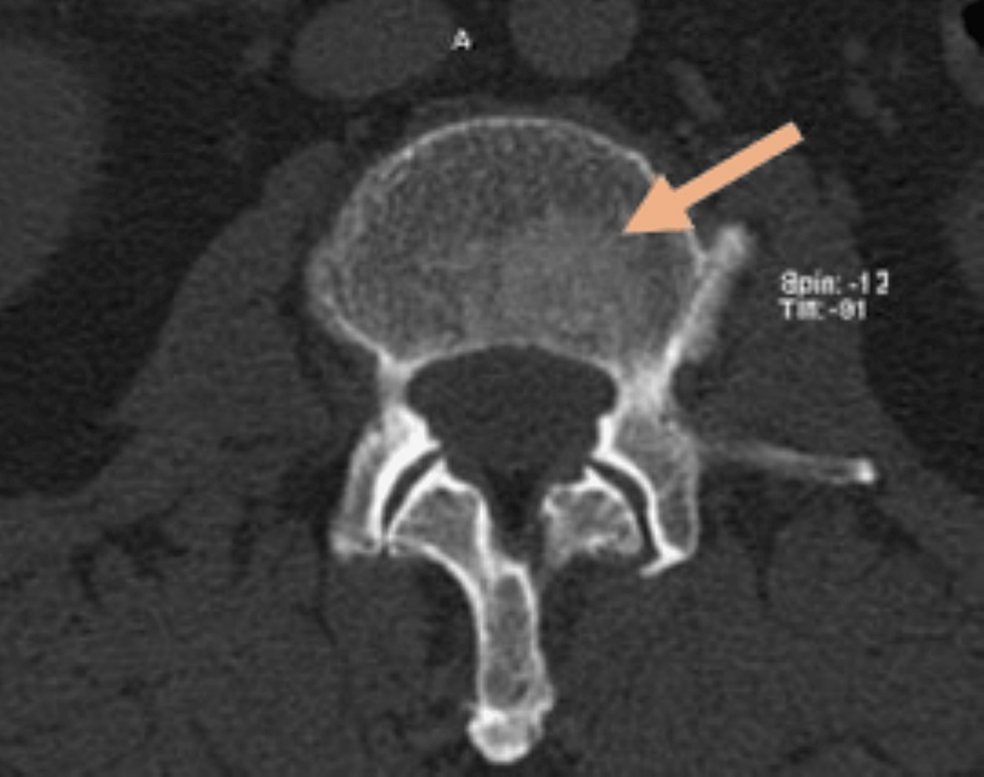
Cross-section of a computed tomography image of the L3 vertebra, in which an osteoblastic lesion is evident (orange arrow).

**Figure 2:**
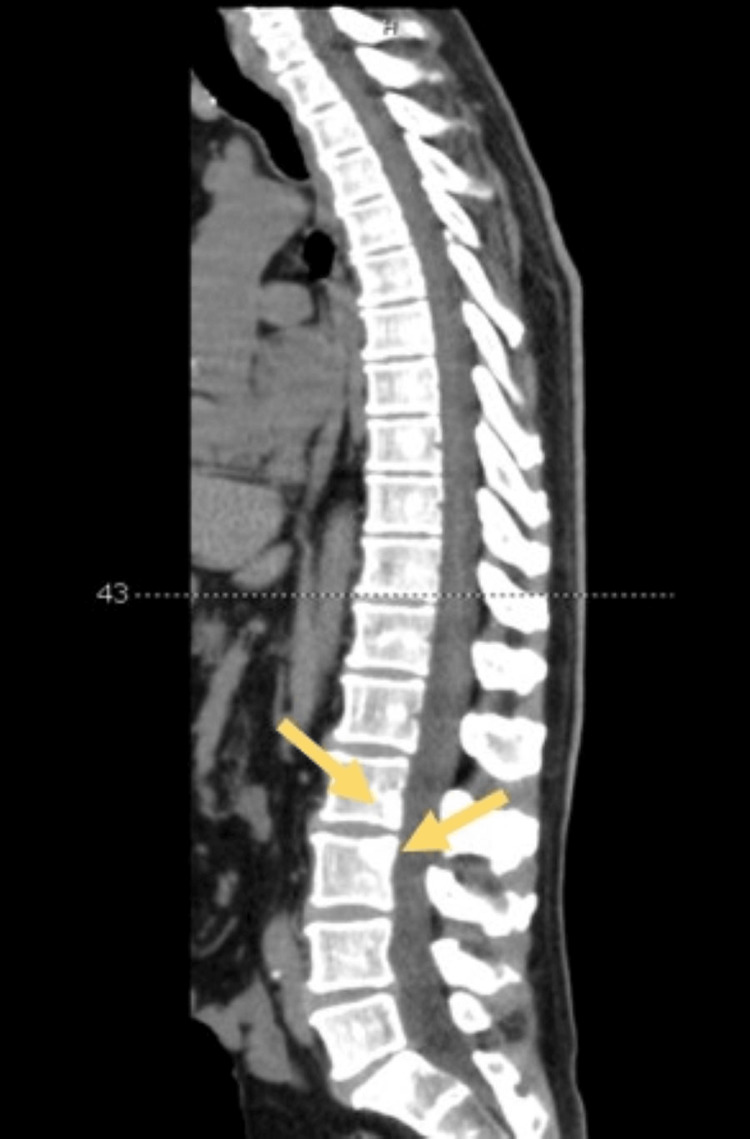
Sagittal section of a computed tomography image of the spine, with particular emphasis on osteoblastic lesions in the L2 and L3 vertebrae (yellow arrows).

The initial blood workup (Table 1) showed thrombocytopenia (109 000 platelets/uL) and elevation of lactate dehydrogenase (661 U/L), alkaline phosphatase (473 U/L), and C-reactive protein (179.97 mg/L). Computed tomography (CT) of the spine revealed multiple bone lesions of apparent osteoblastic nature in the lumbar and dorsal spines and iliac bones (Figures 1, 2). Magnetic resonance imaging of the lumbar spine also suggested osteoblastic metastatic infiltration.

**Table 1:** Laboratory findings during the clinical investigation

Laboratory test	Results			Reference Values
	Admission	At 1.5 weeks	At 5 weeks	
White Blood Cells (cells/μL)	10 270	8 150	7 740	4 000 to 10 500
Hemoglobin (g/dL)	14.4	13.3	8.0	13.5 to 18.0
Mean Corpuscular Volume (fL)	86.1	84.8	82.3	78.0 to 100.0
Platelets (cells/μL)	109 000	135 000	22 000	150 to 450
Albumin (g/dL)	3.7	3.2	-	3.4 to 4.8
Sodium (mEq/L)	139	135	140	136 to 145
Potassium (mEq/L)	4.6	4.4	4.2	3.5 to 5.1
Calcium (mg/dL)	-	9.1	-	8.4 to 9.7
Creatinine (mg/dL)	0.8	0.6	0.6	
Urea nitrogen (mg/dL)	45.6	21.9	17.8	0.7 to 1.2
C-reactive protein (mg/L)	179.97	-	321.42	< 5
Lactate dehydrogenase (U/L)	661	561	890	< 250
Alkaline phosphatase (U/L)	473	506	631	40 to 129
Ca 19.9 (U/mL)	-	190.3	-	< 27
CEA (carcinoembryonic antigen) (ng/mL)	-	25.4	-	< 5.5
β2- macroglobulin (μL/L)	2 840	-	-	800 to 2000

Proceeding with the etiological study, a thoracic, abdominal, and pelvic CT scan was performed, which did not show any lesion compatible with a primary neoplasm. In addition, prostatic, scrotal, and thyroid ultrasounds were done with no suspicious lesions found. Nevertheless, an upper gastrointestinal endoscopy revealed a gastric ulcerated lesion with protruding edges in the angular incisure. The anatomopathological (AP) examination of this gastric lesion revealed characteristics of a mixed adenocarcinoma with tubular areas and areas of loosely cohesive cells with a signet ring phenotype.

**Figure 3:**
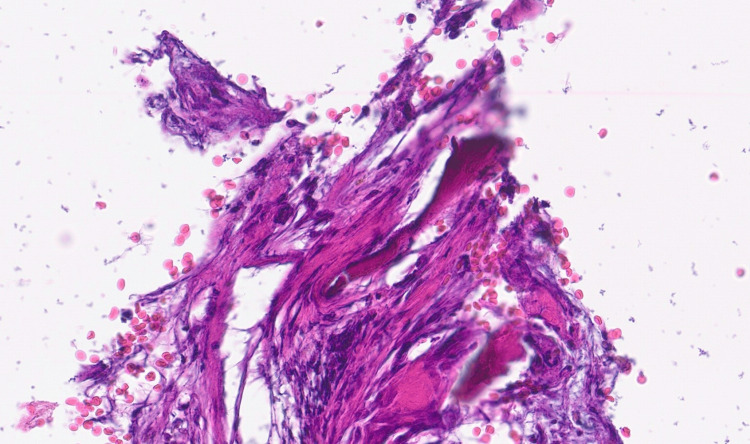
Bone tissue fragment with evidence of neoplastic cells (hematoxylin and eosin stain, 20x).

A biopsy of one of the bone lesions was performed, and the AP examination confirmed the presence of neoplastic metastasis cells (Figure 3), with a positive immunohistochemistry study for the expression of cytokeratin AE1/AE3 and a negative for prostate-specific antigen, making this osteoblastic lesion compatible with metastatic tissue originating from metastatic gastric adenocarcinoma. 

The case was discussed in a multidisciplinary oncological therapeutic decision consultation (MOTDC), and it was established to perform a medullogram to exclude medullary invasion (given the type of lesions and worsening of bicytopenia) and, thus, decide on the definitive therapy to be carried out.

A myelogram was also performed, showing no alterations. Concerning tumor markers, there was an increase in β2- microglobulin, Ca 19.9, and carcinoembryonic antigen (CEA) (Table 1).

Considering the finding of an ulcerated gastric lesion compatible with a mixed-type gastric adenocarcinoma, the identification of osteoblastic bone lesions compatible with secondary lesions from the metastatic gastric adenocarcinoma, and also the exclusion of other primary neoplasmic lesions, namely prostatic and pulmonary, the diagnosis of stage IV mixed-type gastric adenocarcinoma was established. 

One month after the admission to the emergency department, just a few days after the MOTDC, the patient presented with left homonymous hemianopsia and was diagnosed with an ischemic stroke involving the right posterior cerebral artery territory. At this time, concerning the tumor, no systemic therapy had already been initiated. The blood workup had already shown normocytic anemia, with a hemoglobin value of 8.0 g/dL, severe thrombocytopenia, and worsening of alkaline phosphatase, lactate dehydrogenase, and C-reactive protein values (Table 1). The patient died one week later, corresponding to a five-week period after the first complementary examination to suggest osteoblastic lesions.

## Discussion

Gastric carcinomas rarely metastasize to the bone and even more rarely with osteoblastic presentation. The presence of bone metastasis in malignant neoplasms of the stomach is usually indicative of the existence of a more extensive metastatic disease with liver and lung involvement preceding or coexisting with these lesions [4].

The incidence of bone metastases from gastric cancer was found to be between 2% and 20%, most frequently presenting as late complications occurring years after diagnosis and/or removal of the primary tumor [1,2]. Some postmortem studies describe skeletal metastases in only 5% of patients with gastrointestinal cancer, and osteoblastic implants for primary gastric cancer are considered a very rare finding [5].

To the authors knowledge, until now, only 13 cases of osteoblastic bone metastases have been associated with signet ring cell gastric carcinoma. However, our patient presents as the first case of mixed-type gastric adenocarcinoma (with both signet ring cells and tubular cell components) with osteoblastic metastases associated, which was the patient’s first presentation.

Bone metastases, as the first presentation of gastric cancer without gastrointestinal symptoms, are very rare and known to be associated with a very poor prognosis, as it happened to our patient. He survived only five weeks from the first complementary diagnostic test showing bone lesions and about three and a half weeks after the gastric lesion biopsy, it not being perfectly clear if the death was either due to the progression of the metastatic tumor or to the stroke consequences (itself a probable complication associated with the neoplasia), but most likely to a combination of both. In fact, in the published literature, some studies reported a median survival time, after detection of bone involvement, of 4 to 6 months [3,6-9].

An insidious or even asymptomatic presentation of a gastric carcinoma may lead to a significant number of diagnoses already in the advanced stages of the neoplasic disease, compromising its treatment and prognosis since, in the presence of distant metastases, gastrectomy is only considered a palliative therapeutic measure used to prevent tumor-related complications [4].

Our patient’s presentation with severe lower back pain and weight loss but no gastrointestinal symptoms led to the identification of osteoblastic lesions before the primary tumor. Therefore, despite the diagnostic challenge, after excluding other much more common primary tumor locations, an ulcerated gastric lesion was identified in the endoscopy carried out during the exhaustive study of occult primary neoplasia, leading to the diagnosis of gastric adenocarcinoma.

Also, the presence of elevated tumor markers such as Ca 19.9 and CEA could have helped to direct this diagnosis, even if the association between osteoblastic lesions and gastric tumors is very rare [5].

Concerning treatment, in patients with advanced metastatic disease, the main goals to aim for are to alleviate symptoms, improve quality of life, and increase the patient's survival. In these patients, systemic therapy with chemotherapy is the first-line treatment, with or without the combination of targeted therapy and immunotherapy.

However, a multidisciplinary approach is often necessary in the management of other symptoms and complications, such as nausea, pain, obstruction, perforation, or bleeding. In these cases, it may be necessary to resort to endoscopic treatment, radiotherapy, or even gastrectomy (not recommended in general but reserved for very specific cases, namely in patients with good functional status) [4,10].

## Conclusions

The authors presented this case of a 61-year-old man with a diagnosis of mixed-type gastric adenocarcinoma, whose first finding was osteoblastic lesions, with a very unfavorable evolution and outcome and a survival time much lower than the median survival published in the few cases available in the literature. This case was presented not only because of its complexity and rarity but also to highlight that, despite the fact that the association between gastric adenocarcinomas and osteoblastic metastasis is extremely rare, and its presentation might even be insidious and unclear, it should be considered in the etiological study of a primary tumor in patients with secondary bone lesions.
